# Endothelial function measured by peripheral arterial tonometry in patients with chronic myeloid leukemia on tyrosine kinase inhibitor therapy: a pilot study

**DOI:** 10.1186/s40959-023-00164-3

**Published:** 2023-02-22

**Authors:** Tomohiro Kaneko, Sakiko Miyazaki, Azusa Kurita, Ryoko Morimoto, Shun Tsuchiya, Naoki Watanabe, Tomoiku Takaku, Norio Komatsu, Tohru Minamino

**Affiliations:** 1 Department of Cardiovascular Biology and Medicine, Juntendo University Graduate School of Medicine, 2-1-1 Hongo, Bunkyo-ku, Tokyo Japan; 2 Department of Hematology, Juntendo University Graduate School of Medicine, 2-1-1 Hongo, Bunkyo-ku, Tokyo Japan

**Keywords:** Tyrosine kinase inhibitors, Chronic myeloid leukemia, Endothelial dysfunction, Uric acid

## Abstract

**Background:**

Arterial occlusive events are an emerging problem in patients with chronic myeloid leukemia (CML) receiving tyrosine kinase inhibitor (TKI) therapy. Endothelial cell damage is thought to play an important role in the development of vascular events. Measurement of the peripheral vasodilator response by peripheral arterial tonometry (PAT) has reportedly been useful in the non-invasive assessment of endothelial dysfunction. To date, no studies have assessed endothelial function using PAT in patients with CML receiving TKIs.

**Method:**

We measured the reactive hyperemia index (RHI) using PAT in young patients with CML (men aged ≤ 55 years and women aged ≤ 65 years) receiving TKIs.

**Results:**

Thirty patients with CML were examined (mean age, 43.5 ± 9.8 years; men, 57%). The median RHI was 1.81. Among these patients, 16.7% and 83.3% were taking imatinib and second- or third-generation TKIs, respectively. There were no differences in the baseline characteristics between the low RHI (< 1.67, *n* = 10), borderline RHI (≥ 1.67 and < 2.10, *n* = 14), and normal RHI (≥ 2.10, *n* = 6) groups. Serum uric acid (UA) levels and the RHI were significantly negatively correlated (*r* = -0.40, *p* = 0.029).

**Conclusion:**

One-third of young patients with CML receiving TKI therapy were classified as having a low RHI. The RHI was negatively correlated with serum UA level. Larger prospective studies are necessary to examine whether the RHI predicts cardiovascular events in such patients.

## Introduction

Tyrosine kinase inhibitors (TKIs) have dramatically improved the prognosis of patients with chronic myeloid leukemia (CML). Imatinib, a first-generation BCR-ABL TKI, was approved for the treatment of CML in 2001. Currently second- (dasatinib, nilotinib, and bosutinib) and third-generation (ponatinib) TKIs are available for CML treatment and have showed rapid and deeper molecular responses (MRs) than imatinib. Four TKIs other than ponatinib are approved for first-line treatment and ponatinib is approved for patients with T315I mutations, which result in resistance to treatment with other TKIs [1–5]. Therefore, treatment-free remission following TKI cessation is an emerging goal for patients with CML for whom TKI use has yielded a deep and stable molecular response (patient who achieved MR4.5 and sustained 2 years). However, about half of patients relapsed within 2 years after cessation of TKI therapy in previous studies [5–7]. The management of various adverse events (AEs) has become a major issue in clinical practice. These include cardiovascular adverse events (CAEs), such as ischemic heart disease, cerebral infarction, and peripheral artery occlusive disease [8].

CAEs have been reported not only in patients with multiple risk factors for atherosclerosis, but also in young patients with CML who are considered to be at low risk [8]. In particular, nilotinib, ponatinib, and dasatinib are prone to result in CAEs. Incident rates among patients treated with nilotinib were 10.6% at 5 years and 24.8% at 10 years in one study [1]. The 5-year cumulative incidence rates of arterial occlusive events (including cardiovascular, cerebrovascular, and peripheral vascular events) in patients with CML treated with ponatinib and dasatinib were 31% [2] and 7%, respectively [5]. Although the pathophysiology of drug-induced CAEs remains unclear, several in vitro studies have revealed an effect of TKI on vascular endothelial cells (ECs) [9–11]. Hence, we hypothesized that various drug-induced effects on ECs may have roles to play in the development of CAEs in patients with CML treated with TKIs. However, an appropriate method and biomarker for the evaluation of EC functions has not been established.

Reactive hyperemia peripheral arterial tonometry (RH-PAT) using the EndoPAT 2000 system (Itamar Medical Inc., Caesarea, Israel) is a well-known non-invasive method for evaluation of the vascular endothelial function [12]. Furthermore, the reactive hyperemia (RH) response, as measured with the reactive hyperemia index (RHI), was an independent predictor of cardiovascular events beyond the traditional Framingham risk score during a 7-year follow-up study [13]. To date, no studies have investigated the clinical indicators of vascular endothelial function in patients with CML treated with TKI. This study aimed to evaluate the endothelial function using RH-PAT in patients with CML receiving TKI therapy and to assess the correlation between the RHI and multiple clinical factors.

## Methods

### Patient selection

This was a single-center, cross-sectional study. Consecutive patients with CML on TKI therapy who visited the hematology department of Juntendo University Hospital between January 2020 and July 2021 were considered eligible for this study. The inclusion criteria were men aged ≤ 55 years and women aged ≤ 65 years. We adopted these age thresholds for what is considered “premature atherosclerotic cardiovascular disease” as defined in the guidelines on cardiovascular disease [14]. Written informed consent was obtained from all patients. The Institutional Review Board of Juntendo University Hospital approved the study protocol, and all aspects of the study complied with the Declaration of Helsinki.

### Data collection

Clinical data were collected from the patients’ medical records. We used blood sample data acquired on the day closest to the RH-PAT study within 30 days. We collected arterial blood pressure measurements at the time of the RH-PAT study.

The MR was assessed by quantitative reverse transcription polymerase chain reaction using peripheral blood on the International Scale (IS). We defined the major molecular response (MMR) (≤ 0.1% BCR-ABL on the IS) and deep MR (DMR) (≤ 0.01% BCR-ABL on the IS) according to the 2020 European LeukemiaNet recommendations [15, 16].

### Endothelial function measurement

**Fig. 1:**
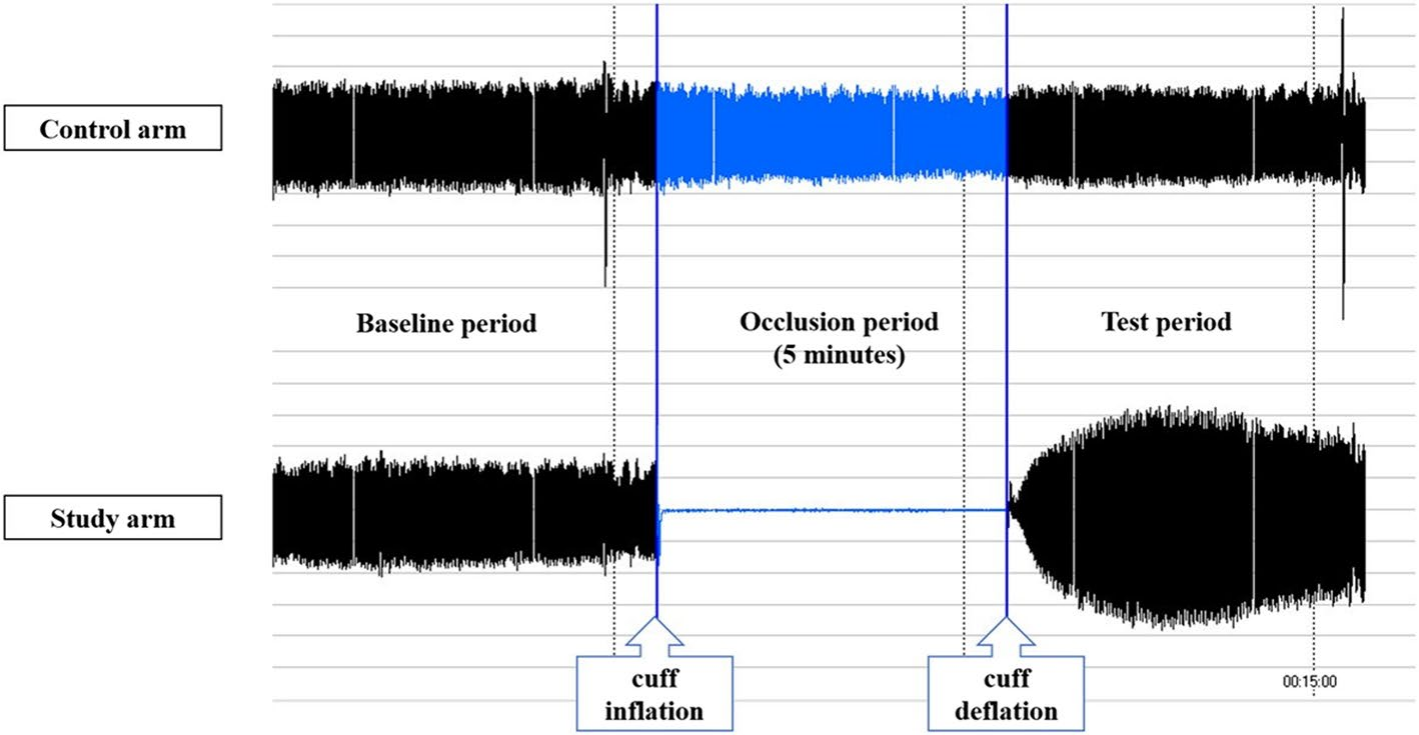
Representative reactive hyperemia peripheral arterial tonometry (RH-PAT) recording. Normal response is characterized by a significant increase in the signal amplitude in the test arm after cuff deflation compared with that at baseline

We used the EndoPAT 2000 system for RH-PAT to evaluate endothelial function. The detailed principles and measurement procedures were previously described [17]. RH-PAT was performed early in the morning with the patient on an empty stomach, in a quiet room with a steady room temperature. Patients were instructed to avoid their daily antihypertensive medication on the day of measurement. After 15 min of bed rest, the PAT probe was positioned on the second finger of each hand. After 5 min of baseline PAT in both hands, the blood pressure cuff was inflated on the test arm to 60 mmHg above the patient’s systolic pressure or at least 200 mmHg for 5 min, and PAT was performed for 5 min after cuff deflation. The increase in pulse amplitude in the hyperemic finger was recorded and analyzed, using an automated operator-independent algorithm, as the RHI (Fig. 1).

### Statistical analysis

Data are presented as mean ± SD or median [interquartile range, IQR] values for continuous variables and as frequencies (%) for categorical variables. Qualitative data were compared using the chi-square or Fisher's exact probability test, and continuous variables were compared using the one-way analysis of variance (ANOVA). Pearson’s correlation coefficient or Spearman’s rank correlation coefficient was used to determine the relationship between clinical factors and the RHI. Stepwise multiple linear regression analysis was also performed to determine the independent determinants of the RHI. Multiple linear regression analysis included variables with a *p*-value < 0.1 in the correlation analysis. ANOVA *p*-value and coefficient of determination (*R*^2^) for regression analysis were shown. All analyses were conducted using SPSS Statistics 21.0 (IBM, Armonk, New York). Statistical significance was set at a two-tailed *p*-value of < 0.05.

## Results

**Fig. 2:**
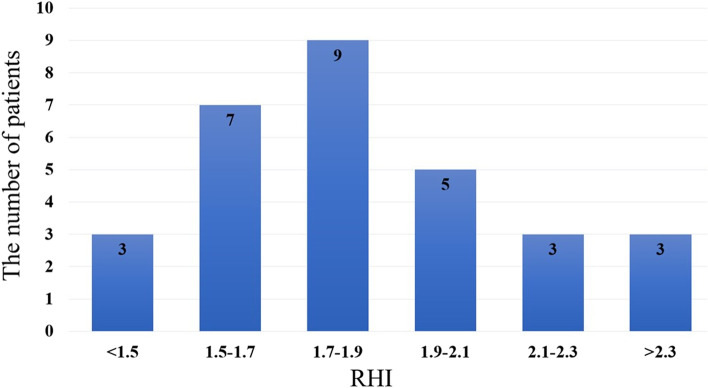
Distribution of the reactive hyperemia index (RHI)

A total of 30 patients were included in this study. Table 1 shows the baseline characteristics of the patients. The mean age was 43.5 ± 9.8 years, 57.0% were men, 20.0% had hypertension, 53.3% had dyslipidemia, and 23.3% were current smokers. None of the patients were taking beta-blockers. The median duration of TKI therapy was 1466 [980–2256] days, and the median RHI was 1.81 [1.6–2.05]. In terms of TKI selection, 16.7% used imatinib, 40.0% used nilotinib, 13.3% used dasatinib, 16.7% used bosutinib, and 13.3% used ponatinib. In total, 18/30 (60.0%) achieved a DMR and 9/30 (30.0%) achieved an MMR. Figure 2 shows the distribution of the RHI in the study patients. Only six patients (20%) were within the normal range (RHI ≥ 2.10) [18].

**Table 1:** Baseline characteristics

N		**RHI**			
	**All**	**Low RHI** **(< 1.67)**	**Borderline RHI** **(≥ 1.67 and < 2.1)**	**Normal RHI** **(**≥ **2.1)**	***P*-value**
	***N* = 30**	***N* = 10**	***N* = 14**	***N* = 6**	
Age, yrs	43.5 ± 9.8	42.6 ± 9.5	44.4 ± 11.6	43.0 ± 6.4	0.908
Male/Female, n (%)	17/13 (57/43)	8/2 (80/20)	6/8 (43/57)	3/3 (50/50)	0.181
Height, cm	166.3 ± 8.7	168.1 ± 9.0	163.6 ± 8.5	170.0 ± 7.8	0.252
Weight, kg	67.5 ± 13.6	69.9 ± 13.2	63.3 ± 12.4	73.2 ± 15.9	0.265
BMI, kg/m^2^	24.3 ± 3.7	24.7 ± 4.0	23.5 ± 2.8	25.4 ± 4.9	0.535
Systolic BP, mmHg	122.6 ± 21.9	122.4 ± 17.1	128.1 ± 24.2	110.0 ± 21.0	0.245
Diastolic BP, mmHg	69.4 ± 15.6	74.0 ± 11.0	70.6 ± 15.5	58.7 ± 19.4	0.149
Current smoker, n (%)	7 (23.3)	5 (50.0)	2 (14.3)	0 (0)	0.151
Former smoker, n (%)	6 (20.0)	1 (10.0)	3 (21.4)	2 (33.3)	0.151
Hypertension, n (%)	6 (20.0)	2 (20.0)	3 (21.4)	1 (16.7)	0.971
Dyslipidemia, n (%)	16 (53.3)	5 (50.0)	8 (57.1)	3 (50.0)	0.926
Diabetes mellitus, n (%)	3 (10.0)	2 (20.0)	1 (7.1)	0 (0)	0.386
MMR, n (%)	9 (30.0%)	2 (20.0%)	4 (28.6%)	3 (50.0%)	0.683
DMR, n (%)	18 (60.0%)	7 (70.0%)	8 (57.1%)	3 (50.0%)	0.683
**Current medication**					
TKI Imatinib	5 (16.7)	1 (10.0)	2 (14.3)	2 (33.3)	0.151
Nilotinib	12 (40.0)	3 (30.0)	8 (57.1)	1 (16.7)	
Dasatinib	4 (13.3)	1 (10.0)	2 (14.3)	1 (16.7)	
Bosutinib	5 (16.7)	2 (20.0)	2 (14.3)	1 (16.7)	
Ponatinib	4 (13.3)	3 (30.0)	0 (0)	1 (16.7)	
TKI duration, days	1466 [980–2256]	1643 [1213–2160]	1352 [925–2128]	1354 [1074–2370]	0.830
Statin, n (%)	12 (40.0)	3 (30.0)	7 (50.0)	2 (33.0)	0.574
ACE-I/ARB, n (%)	5 (16.7)	1 (10.0)	3 (21.4)	1 (16.7)	0.760
CCB, n (%)	5 (16.7)	1 (10.0)	3 (21.4)	1 (16.7)	0.760
Xanthine oxidase inhibitor, n (%)	3 (10.0)	2 (20.0)	0 (0)	1 (16.7)	0.227
**Laboratory data**					
WBC, × 10^3^/µL	6.5 ± 1.9	6.9 ± 1.9	6.7 ± 2.1	5.4 ± 1.5	0.277
Hemoglobin, g/dL	13.6 ± 1.7	14.6 ± 1.7	13.3 ± 1.9	12.8 ± 1.5	0.082
Serum creatinine, mg/dL	0.78 ± 0.25	0.84 ± 0.17	0.77 ± 0.32	0.72 ± 0.18	0.642
Serum uric acid, mg/dL	5.0 ± 1.2	5.7 ± 0.9	4.8 ± 1.2	4.5 ± 1.5	0.077
Triglycerides, mg/dL	83 [56–116]	74 [56–163]	72 [52–101]	98 [78–169]	0.243
HDL-C, mg/dL	61 ± 16	64 ± 20	62 ± 14	53 ± 15	0.450
LDL-C, mg/dL	110 ± 28	104 ± 29	122 ± 22	92 ± 32	0.068
HbA1c, %	5.6 ± 0.58	5.8 ± 0.86	5.5 ± 0.35	5.6 ± 0.28	0.362

*BMI* Body mass index, *BP* Blood pressure, *MMR* Major molecular response, *DMR* Deep molecular response, *TKI* Tyrosine kinase inhibitor, *CCB* Calcium channel blocker, *ACE-I/ARB* Angiotensin converting enzyme inhibitor/angiotensin II receptor blocker, *WBC* White blood cell, *HDL-C* High-density lipoprotein cholesterol, *LDL-C* Low-density lipoprotein cholesterol

**Fig. 3:**
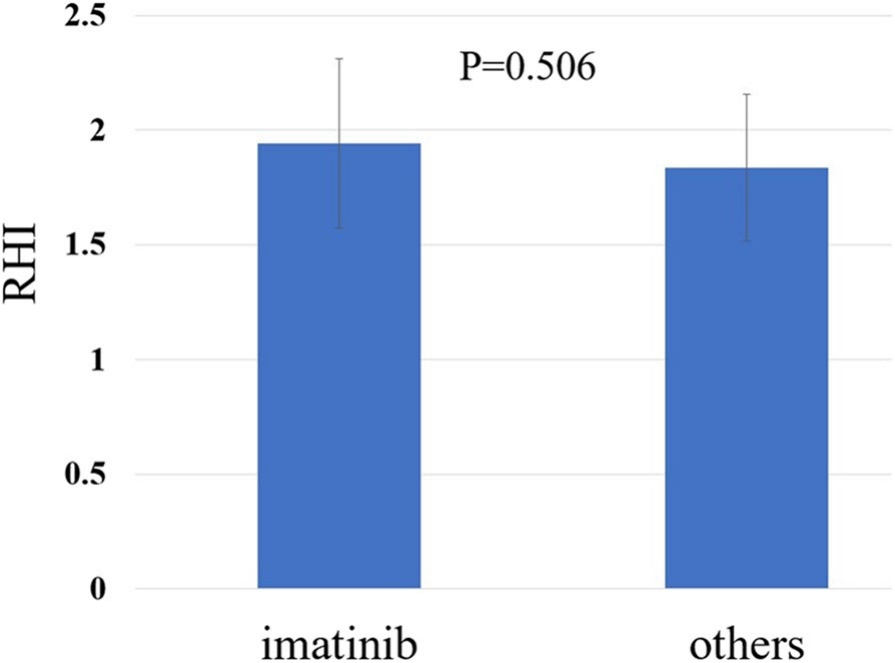
Comparison of the reactive hyperemia index (RHI) between patients receiving imatinib and those receiving second- or third-generation TKIs. No difference is seen between imatinib and newer TKIs in terms of the RHI

**Fig. 4:**
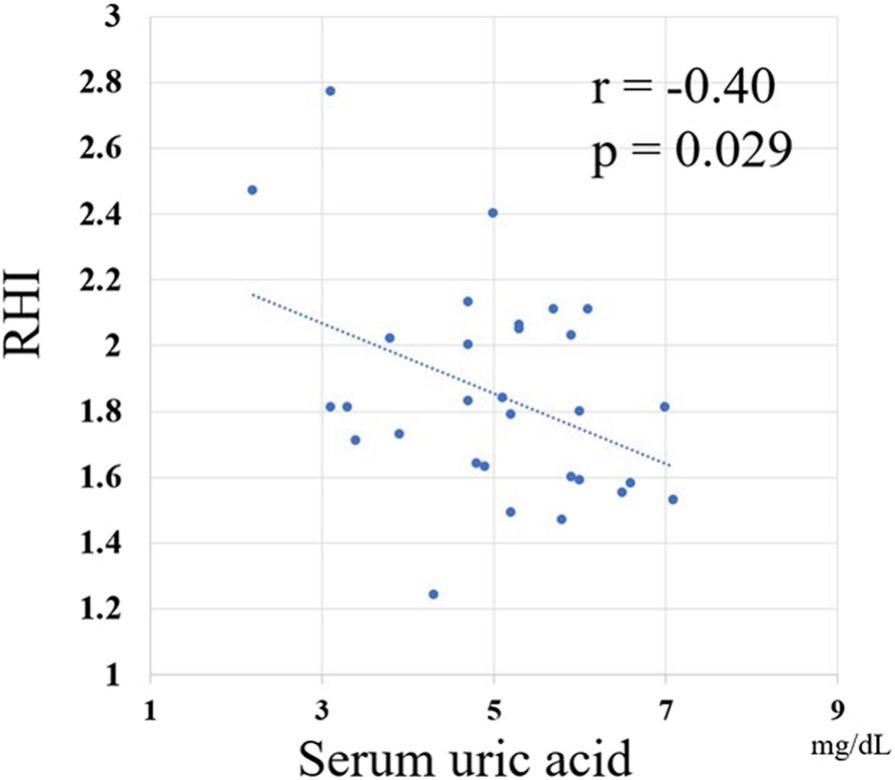
Correlation between the reactive hyperemia index (RHI) and serum uric acid (UA) levels. The RHI is significantly correlated with serum uric acid (UA) levels

Patients were divided into three groups based on the RHI cutoff value proposed by Tanaka et al. [17]: low (< 1.67), borderline (≥ 1.67 and < 2.10), and normal (≥ 2.10) RHI groups. There were no differences in treatment responses among the three groups. Patients in the normal-RHI group (*n* = 6) tended to have lower hemoglobin levels, low-density lipoprotein (LDL) cholesterol levels, and serum uric acid (UA) levels than those in the low-RHI group (*n* = 10); however, these differences were not statistically significant. Selected TKIs in each group were similar between the normal and low-RHI groups (Table 1). There was also no difference between imatinib and newer generation TKIs in terms of the RHI (Fig. 3). In univariate analysis, the RHI was significantly correlated with diastolic blood pressure (*r* = -0.393, *p* = 0.032) and serum UA levels (*r* = -0.399, *p* = 0.029) (Table 2, Fig. 4). In the multiple linear regression model, serum UA levels were inversely associated with the RHI (*p* = 0.029) (Table 3).

**Table 2:** Univariate analysis of correlation between RHI and clinical indicators

Variables	r	*P*-value
Age, yrs	0.139	0.464
Male sex	-0.222	0.239
BMI, kg/m^2^	0.013	0.945
Systolic BP, mmHg	-0.197	0.297
Diastolic BP, mmHg	-0.393	0.032
Current smoker	-0.36	0.051
Hemoglobin, g/dL	-0.356	0.053
Serum creatinine, mg/dL	-0.141	0.458
Serum uric acid, mg/dL	-0.399	0.029
Triglycerides, mg/dL	0.070	0.713
HDL-C, mg/dL	-0.163	0.390
LDL-C, mg/dL	0.025	0.894
HbA1c, %	-0.118	0.534

*BMI* Body mass index, *BP* Blood pressure, *TKI* Tyrosine kinase inhibitor, *CCB* Calcium channel blocker, *ACE-I/ARB* Angiotensin converting enzyme inhibitors/angiotensin II receptor blockers, *WBC* White blood cell, *HDL-C* High-density lipoprotein cholesterol, *LDL-C* Low-density lipoprotein cholesterol

**Table 3:** Multiple linear regression analysis (dependent variable: RHI)

	**Non-standardized coefficients (B)**	**Standardized coefficients (β)**	***P* value**
Constant	2.388		< 0.0001
Serum uric acid, mg/dL	-0.107	-0.399	0.029

Variables *p* < 0.1 in correlation analysis (diastolic blood pressure, smoker, hemoglobin, and uric acid) were included in the analysis. Overall *R*^2^ = 0.16, ANOVA *p* = 0.029

## Discussion

To the best of our knowledge, this is the first study to evaluate vascular endothelial function using non-invasive RH-PAT in patients with CML receiving TKIs. Only 20% of the patients in this study exhibited normal RHI levels. Even in younger patients, who are generally considered to be at low risk, vascular endothelial function may have been impaired during TKI therapy. In univariate analysis, the RHI was significantly correlated with diastolic blood pressure and serum UA levels. Multiple regression analysis showed that serum UA levels were independently associated with the RHI. Because this is a cross-sectional study, it is unknown whether the RHI value can be a predictor of cardiovascular events in patients with CML; however, we believe that the findings from this pilot study will be useful for conducting prospective trials in the future.

### TKI-associated arterial occlusive events in CML—the difference between first and newer generation TKIs

All TKIs approved for CML treatment share kinase inhibition activity against BCR-ABL; however, they differ in their kinase inhibition profiles (the so-called “off-target effect”), and some are vascular biology-related kinases [9]. Several reports have revealed the direct effects of TKIs on vascular ECs. For example, nilotinib upregulates pro-atherogenic adhesion-proteins (ICAM-1 and VCAM-1), which induces atherosclerosis on the cell surface [10]. In addition, Grover-Proakter et al. reported that nilotinib, dasatinib, and ponatinib induced more than 100 gene expression changes of human umbilical vein ECs compared to imatinib and bosutinib treated cells. Moreover, decreased tube formation and low cell viability were observed in human umbilical vein ECs treated with these three TKIs [19]. However, an appropriate method for evaluating EC function and damage during TKI therapy is lacking. Flow-mediated vasodilation (FMD) is a well-established method for assessing endothelial function and has been reported to be associated with cardiovascular events that develop during the treatment of multiple myeloma [20]. In addition, soluble ICAM-1 and endothelial function measured by FMD of the brachial artery are correlated with each other [21]. However, the assessment of brachial artery reactivity using ultrasound is technically challenging and involves a significant learning curve. It is recommended that at least 100 supervised scans and measurements are performed annually [22]. Conversely, PAT is a versatile, alternative method for non-invasive and reproducible assessment of endothelial function; it is also independent of the examiner's skills [12]. Therefore, we used RH-PAT to assess endothelial function in this study. FMD and RH-PAT are based on the same principle of the RH phenomenon and have been reported to significantly predict cardiovascular events with similar prognostic magnitudes; however, no statistically significant relationship was noted between them [23]. These findings suggest that these tests reflect different aspects of vascular function, and future research is required to investigate the differences in the predictability of cardiovascular events during TKI therapy.

In their review, Tanaka et al. [18] classified the RHI into three categories according to the risk of cardiovascular events: normal (≥ 2.10), borderline (≥ 1.67 and < 2.10), and abnormal (< 1.67). In our study, only six patients (20%) had an RHI within the normal range. Furthermore, to investigate the effects of newer generation TKIs (dasatinib, nilotinib, bosutinib, and ponatinib) on endothelial function, the RHI in patients treated with newer generation TKIs was compared with those treated with imatinib; however, the difference was not statistically significant. Given that the number of patients who received imatinib in our study was small, a study with a larger sample is necessary to compare the effects of imatinib with those of the newer generation TKIs on endothelial function.

### UA and endothelial function

Several epidemiological studies have confirmed hyperuricemia as a risk factor for cardiovascular diseases [24, 25]. UA is the end product of purine metabolism and is catalyzed by xanthine oxidoreductase (XOR). Under conditions in which XOR activity is enhanced, UA and reactive oxygen species (ROS) are generated concomitantly. ROS may disrupt endothelial function through the reaction of superoxide (O_2_^−^) with nitric oxide (NO), leading to a decrease in NO bioavailability and increased production of peroxynitrite (ONOO^−^) [26]. Recent clinical studies have shown that FMD is more impaired in patients with hyperuricemia than in those without [27, 28]. Taher et al. showed that normal–increased UA levels were associated with endothelial dysfunction (RHI < 2.0) in women at low risk of cardiovascular diseases [29]. In addition, the serum UA level is associated with the RHI in patients with low-risk hypertension but not in patients with high-risk hypertension [30]. The authors of that report speculated that UA may be an important biomarker for investigating early signs of endothelial dysfunction in low-risk patients.

Early in the development of hematopoietic tumors and immediately after the start of chemotherapy, tumor cells rapidly disintegrate and produce excessive amounts of UA [31]. Tumor lysis syndrome (TLS) is a fulminant form of this pathology; however, the risk of TLS is low in patients with chronic-phase CML receiving TKI therapy [32]. All patients in this study had chronic-phase CML, and few had abnormally high serum UA levels. We found a significant inverse correlation between serum UA levels and the RHI, suggesting that UA affects vascular endothelial function even if it is within normal limits.

### Limitations

This was a single-center, cross-sectional study based on a small number of patients, without any comparisons with healthy subjects. It remains unclear whether the RHI predicts cardiovascular events in patients with CML receiving TKIs. In addition, the differences between the effects of each TKI on endothelial function need to be clarified. To investigate these points, longitudinal studies with larger numbers of patients are required in the future.

## Conclusions

The RHI, a clinical indicator of endothelial function, was negatively correlated with serum UA levels in young patients with CML receiving TKI therapy. One-third of young patients with CML receiving TKIs were classified as having a low RHI, although it was unclear whether the RHI predicted CAEs in these patients. Further research is required to investigate the relationship between drug-induced CAEs and the RHI in patients with CML.

## Data Availability

The datasets used and/or analyzed during the current study are available from the corresponding author on reasonable request.

## Abbreviations

AE: Adverse event
ANOVA: Analysis of variance
CAE: Cardiovascular adverse event
CML: Chronic myeloid leukemia
DMR: Deep molecular response
EC: Endothelial cells
FMD: Flow-mediated vasodilation
IS: International Scale
MMR: Major molecular response
MR: Molecular response
PAT: Peripheral arterial tonometry
RH: Reactive hyperemia
RHI: Reactive hyperemia index
ROS: Reactive oxygen species
TKI: Tyrosine kinase inhibitor
TLS: Tumor lysis syndrome
UA: Uric acid
XOR: Xanthine oxidoreductase
